# Evaluation of Immunogenicity and Cross-Protective Efficacy of a CpG-Adjuvanted Trivalent Inactivated Influenza Vaccine in Ferrets

**DOI:** 10.3390/vaccines14070615

**Published:** 2026-07-14

**Authors:** Yanping Qiu, Yan Zhang, Shuangshuang He, Yutian Wang, Ruixin Wang, Yanxiao Han, Wen He, Eiketus Sho, Shaohua Han, Haojie Wu

**Affiliations:** 1 Huan Ke (Hebei) Biotechnology Co., Ltd., Shijiazhuang 050000, China; zhangyan@huapusjz.com (Y.Z.); heshuangshuang@parrbio.com (S.H.); wangyutian@huapusjz.com (Y.W.); wangruixin@huapusjz.com (R.W.); hanyanxiao@huapusjz.com (Y.H.); hanshaohua@huapusjz.com (S.H.); wuhaojie@parrbio.com (H.W.); 2 Huapu Shijiazhuang Pharmaceutical Co., Ltd., Shijiazhuang 050000, China; 3 Jiangsu KMQ BioTech Inc., Nantong 226000, China; hewen@kcibiotech.com (W.H.); zhuangyongjie@kcibiotech.com (E.S.)

**Keywords:** CpG-TIV, ferret model, immunogenicity, protective efficacy, cross-protection

## Abstract

**Background/Objectives**: Pandemic influenza remains a persistent global threat, and while vaccination is the primary preventive measure, conventional vaccines often induce narrow, strain-specific immunity. This study evaluated the immunogenicity, protective efficacy, and cross-protective potential of a CpG-adjuvanted trivalent inactivated influenza vaccine (CpG-TIV) administered intramuscularly at high and low doses in ferrets. **Methods**: Groups of influenza-seronegative ferrets received two intramuscular injections, 3 weeks apart, of high- or low-dose CpG-TIV or a commercial non-adjuvanted trivalent vaccine. Three weeks after the second immunization (Day 42), serum was obtained, and the ferrets were subsequently challenged intranasally with homologous H1N1 and influenza B viruses, as well as a heterologous drifted H3N2 strain. Clinical signs, body weight, nasal viral load, and lung histopathology were monitored following the viral challenge. **Results**: CpG-TIV induced significantly higher dose-dependent HI and IgG antibodies than the commercial unadjuvanted vaccine. High-dose CpG-TIV markedly reduced weight loss, clinical symptoms, nasal viral load (by up to 99%), and lung pathological damage. Notably, high-dose CpG-TIV provided significant cross-protection against heterologous H3N2, whereas the commercial vaccine showed no protective effect. At Day 42, HI GMTs in the high-dose group reached 500, 254, and 594 against H1N1, H3N2, and B strains, respectively, with a maximal 2.58 log10 reduction in H1N1 viral load. **Conclusions**: High-dose CpG-TIV demonstrates strong immunogenicity and robust dose-dependent homologous and heterologous cross-protection in ferrets. The combination of a CpG adjuvant and high-dose antigen broadens protection against drifted influenza viruses, overcoming the narrow coverage of conventional vaccines. These data support further clinical development of this broad-spectrum influenza vaccine candidate.

## 1. Introduction

Influenza is an important acute viral respiratory infectious disease caused by influenza viruses. Influenza viruses belong to the Orthomyxoviridae family and are classified into four types: A, B, C, and D [1]. Currently, seasonal epidemics in humans are caused by H1N1 and H3N2 subtypes of type A and B-Victoria lineage of type B [1,2]. The World Health Organization (WHO) estimates that influenza causes 3–5 million severe cases and 290,000–650,000 respiratory disease-related deaths worldwide each year [2,3,4].

As a major respiratory pathogen causing global seasonal epidemics, influenza viruses result in millions of infections and hundreds of thousands of deaths annually, posing a serious threat especially to the elderly, infants, and immunocompromised populations [1,3]. The high mutation rate and continuous antigenic drift and shift of influenza viruses are the core bottlenecks restricting the protective efficacy of licensed seasonal vaccines [1,5]. Traditional seasonal trivalent or tetravalent inactivated vaccines mainly induce strain-specific humoral immune responses targeted at predicted epidemic strains. Once antigenic mismatch occurs between the vaccine strain and the circulating epidemic strain, protective efficacy drops sharply, often to less than 30% in clinical practice [6,7]. For example, during the 2014–2015 influenza season, due to antigenic drift of H3N2 subtype viruses, the protective rate of commercial vaccines against this subtype was less than 30% in many parts of the world, highlighting the urgent clinical demand for next-generation influenza vaccines with enhanced cross-protective capacity against drifted variants.

Nevertheless, commercially available conventional trivalent inactivated influenza vaccines (TIVs) have obvious clinical limitations. Unadjuvanted seasonal TIVs mainly induce narrow, strain-specific neutralizing antibodies and rarely trigger effective cross-reactive humoral or cellular immunity, resulting in poor protection against antigenically drifted strains [7,8,9]. In particular, the protective effectiveness of traditional TIVs against drifted H3N2 strains is frequently below 30%, failing to provide reliable population-level protection. This long-standing clinical challenge underscores the urgent need to develop innovative vaccines that can induce broader, cross-reactive immune responses beyond strain-specific protection [10,11].

As a potent TLR9 agonist, CpG oligodeoxynucleotide adjuvant can strongly activate both humoral and cellular immune pathways, enhance antigen presentation, promote the production of cross-reactive antibodies, and stimulate T cell-mediated immune responses, thereby effectively broadening the protective spectrum of influenza vaccines [12]. Compared with conventional unadjuvanted vaccines, CpG-adjuvanted vaccines are expected to overcome the narrow protection and poor cross-protection of standard seasonal vaccines, making them an ideal strategy for developing broad-spectrum influenza vaccines [13,14,15].

Ferrets represent the gold standard animal model for influenza virus research, owing to their close resemblance to humans in respiratory tract anatomy, pathological progression following viral infection, and patterns of immune responsiveness [16,17,18]. Accordingly, ferrets have been widely used in preclinical evaluation of the immunogenicity and protective efficacy of influenza vaccines [19,20,21]. However, preclinical evaluations in ferrets have demonstrated that adjuvanted vaccines can overcome this limitation; for instance, stockpiled pre-pandemic H5N1 vaccines adjuvanted with AS03 provided cross-protection against a heterologous H5N2 clade 2.3.4.4 virus challenge, indicating that the ferret model is capable of discriminating broadly protective formulations [19]. Currently available TIVs can induce specific antibodies against vaccine strains in ferrets, but their cross-protective effect against heterologous variant strains is limited [22,23]. Although candidate vaccines, such as novel adjuvanted vaccines and multi-epitope vaccines, have shown potential in cross-protection, most are still in the early stages of development [13,24]. Notably, studies in ferrets have shown that the dose of challenge virus significantly influences shedding outcomes and sensitivity to antiviral treatment [25], underscoring the importance of dose optimization in challenge models [26,27]. However, research on the correlation between “dose-dependent protection” and “heterologous cross-protection” is insufficient [13,14].

Against this background, the present study was designed to systematically evaluate the immunogenicity, homologous protective efficacy, and heterologous cross-protective potential of a novel CpG-adjuvanted trivalent inactivated influenza vaccine containing H1N1 A/Victoria/4897/2022, H3N2 IVR 228, and B/Austria/1359417/2021 strains in the ferret model. The vaccine was tested at high and low doses, with a commercial TIV as the control. The key objectives were to clarify the dose–response relationship of the candidate vaccine, verify its cross-protection against the heterologous drifted H3N2 strain A/Hong Kong/4801/2014, and explore whether CpG adjuvant combined with dose optimization can significantly improve broad-spectrum protective efficacy. This study aims to provide robust preclinical evidence for the rational design and clinical development of broad-spectrum influenza vaccines capable of addressing antigenic drift and mismatch.

## 2. Materials and Methods

### 2.1. Experimental Animals

Seventy-five female ferrets, 5–6 months old, were purchased from Wuxi Coral Reef Biotechnology Co., Ltd. (Wuxi, China), with an animal use license number of SYXK (Su) 2023-0094. After receipt, the animals underwent a 7-day quarantine and acclimation period and were included in the experiment after passing the quarantine. Inclusion criteria: The supplier provided a routine influenza virus subtype detection report, and the hemagglutination inhibition titer against the three challenge strains was negative before enrollment [17,18].

### 2.2. Experimental Materials

#### 2.2.1. Test Articles

Three trivalent split inactivated influenza vaccines were applied in this ferret study, including one commercial seasonal vaccine and two CpG-adjuvanted investigational vaccines manufactured by Huapu Shijiazhuang Pharmaceutical Co., Ltd., Shijiazhuang, China.

Commercial TIV split vaccine (Batch No. 20240512): Formulated to contain 15 μg hemagglutinin (HA) of each strain (A/H1N1, A/H3N2, B Victoria lineage) per 0.5 mL human dose in accordance with the manufacturer’s specifications; CpG-adjuvanted high-dose TIV (CpG-TIV-High, internal test batch No. 20241002-H); CpG-adjuvanted low-dose TIV (CpG-TIV-Low, internal test batch No. 20241003).

For the two investigational vaccine candidates, the HA concentration of each monovalent virus bulk was quantified via single radial immunodiffusion (SRID) following the standard laboratory protocol issued by the World Health Organization [28]. All trivalent investigational formulations were standardized to contain 15 μg HA per viral strain in each 0.5 mL injection volume.

The CpG oligodeoxynucleotide adjuvant HP007 (patent number CN201880025806.2; fully phosphorothioate-modified sequence: 5′-TCGCGAACGTTCGCCGCGTACGTACGCGG-3′) was provided by Huapu Shijiazhuang Pharmaceutical Co., Ltd. Two adjuvant concentration gradients were designed: CpG-TIV-High contained 0.32–0.48 mg HP007 per 0.5 mL dose, while CpG-TIV-Low contained 0.13–0.19 mg HP007 per 0.5 mL dose. An unadjuvanted trivalent split inactivated influenza vaccine (15 μg HA per strain, without CpG adjuvant) was prepared as vaccine control.

All vaccines were stored at 2–8 °C before use.

#### 2.2.2. Viruses and Cells

The following influenza challenge strains were used:

H1N1: A/Victoria/4897/2022 (22/316); H3N2: A/Darwin/9/2021 IVR-228 (21/246); and BV type: Influenza B/Austria/1359417/2021 (22/204), all purchased from NIBSC, Hertfordshire, UK. An additional H3N2 strain, A/Hong Kong/4801/2014 (VR1990), was obtained from ATCC, Manassas, VA, USA. Madin-Darby canine kidney (MDCK) cells were purchased from Suzhou Rui’ang Scientific Instruments Co., Ltd., Suzhou, China.

#### 2.2.3. Main Reagents and Instruments

Cell culture was performed using DMEM medium supplemented with fetal bovine serum and penicillin–streptomycin (Gibco, Grand Island, NY, USA). Hemagglutination inhibition (HI) assays were conducted with turkey red blood cells (Kewei, Yancheng, China) and guinea pig red blood cells (SenBeiJia, Nanjing, China) and receptor-destroying enzyme (RDE, receptor-destroying enzyme; National Institute of Infectious Diseases, Tokyo, Japan). ELISA was carried out using anti-ferret IgG-HRP. RNA extraction, reverse transcription, and quantitative real-time PCR (qRT-PCR) were performed using commercial kits. cDNA was synthesized via PrimeScript™ RT reagent Kit (Takara, Kusatsu, Shiga, Japan). qRT-PCR amplification was completed with Hieff Unicon qPCR TaqMan MIX (Yeasen, Shanghai, China) on a Bio-Rad real-time PCR system (Richmond, CA, USA). All viral experiments were conducted in Class II biosafety cabinets. Optical density was measured using a microplate reader and a spectrophotometer. A complete list of all reagents and instruments with catalog numbers and manufacturers is provided in Supplementary Tables S1 and S2.

### 2.3. Study Design and Experimental Procedures

The experiment was divided into 13 groups (Table 1), with G1-G4 challenged with H1N1 A/Victoria/4897/2022, G5-G8 challenged with H3N2 A/Hong Kong/4801/2014, G9-G12 challenged with Influenza B/Austria/1359417/2021, and G13 as the normal control group (no challenge). Each group had 6 ferrets (3 in the normal group), with specific grouping as follows.

### 2.4. Experimental Procedures

Immunization schedule: All ferrets received intramuscular immunization with a total injection volume of 0.5 mL vaccine or PBS control into the quadriceps muscle of the hind limb. Animals were immunized twice on Day 0 (prime) and Day 21 (booster) with a 3-week interval between two doses; the PBS control group received an equal volume of PBS via the identical administration route.

Challenge method: On Day 43, ferrets were intranasally challenged with influenza virus suspension under light isoflurane anesthesia at a total volume of 1 mL per animal (0.5 mL per nostril).

Sample collection: Peripheral blood was collected on Day 21 and Day 42, and serum was separated for Ig antibody and HAI titer detection; nasal washes were collected on Days 1, 3, and 5 after challenge for virus load detection; animals were euthanized on Day 6 after challenge, and serum was collected and lung tissue was isolated.

Monitoring indicators: From the animals’ entry into the experiment to the end of the experiment, general observations were conducted daily, including cage-side observations of death or moribund state, mental status, behavioral activity, fecal traits, and the supply of feed and water; body temperature and body weight were measured daily starting before challenge, and clinical symptoms were observed and scored according to the standard (Table 2) until the end of the experiment [29].

### 2.5. Detection Methods

#### 2.5.1. Indirect ELISA for Serum Total Ig Antibodies

Serum total Ig antibodies specific for H1N1, H3N2, and BV HA were measured by indirect ELISA using His-tagged recombinant HA proteins: H1N1 (A/Victoria/4897/2022), H3N2 (A/Darwin/9/2021 IVR-228), and BV (B/Austria/1359417/2021). Plates were coated overnight at 4 °C with 100 μL/well of each HA at 1.33 μg/mL in PBS, blocked with 1% BSA for 1 h at 37 °C, and then incubated with 3-fold serially diluted serum (starting at 1:100) for 2 h at 37 °C. Bound antibodies were detected with HRP-conjugated goat anti-ferret Ig H&L, followed by TMB substrate and 1 M H_2_SO_4_ stop solution. Absorbance was read at 450 nm (reference 630 nm). The antibody titer was defined as the reciprocal of the highest dilution with OD_450_ greater than the mean + 3 SD of blank control wells (coated with HA but incubated with sample diluent only). Because the secondary antibody recognizes both heavy and light chains of multiple Ig classes, results are reported as “serum total Ig antibodies”.

#### 2.5.2. Hemagglutination Inhibition Titer Detection

Serum from Days 21 and 42 was treated with receptor-destroying enzyme (RDE) at a serum-to-RDE ratio of 1:3 (*v*/*v*) at 37 °C overnight, heat-inactivated at 56 °C for 30 min, diluted to a final 1:10 with PBS, and adsorbed to remove nonspecific agglutinins. HAI assays were performed using 4 hemagglutination units of the following viruses: A/Victoria/4897/2022 (H1N1), A/Darwin/9/2021 IVR-228 (H3N2), and B/Austria/1359417/2021 (BV). Additionally, Day 42 sera were tested against the heterologous challenge virus A/Hong Kong/4801/2014 (H3N2). Two-fold serially diluted serum (starting at 1:10) was mixed with virus and incubated at room temperature for 30 min. Turkey red blood cells (1%) were added for H1N1 and BV (incubated for 40 min), while guinea pig red blood cells (1%) were used for H3N2 viruses (incubated for 1 h). Positive and negative control sera were included on each plate. The HAI titer was expressed as the reciprocal of the highest serum dilution showing complete inhibition of hemagglutination, defined as a compact RBC button streaming without agglutinated particles upon plate tilting.

#### 2.5.3. Virus Challenge and Viral Load Detection

Three weeks after the booster immunization (Day 42), ferrets were challenged intranasally with 1.0 mL of virus suspension (0.5 mL/nostril) under light isoflurane anesthesia. Challenge viruses and doses: A/Victoria/4897/2022 (H1N1) at 1 × 10^6^ PFU/ferret, A/Hong Kong/4801/2014 (H3N2 drift variant) at 1 × 10^6^ PFU/ferret, and B/Austria/1359417/2021 (BV) at 1 × 10^6^ PFU/ferret. The normal control group received PBS instead of virus and was handled in parallel. Nasal washes were collected on Days 1, 3, and 5 post-challenge; on Day 6, ferrets were euthanized and lung tissues were harvested.

Viral loads in nasal washes were quantified by TCID_50_ assay on MDCK cells. Lung tissue viral loads were determined by both TCID_50_ and qPCR. For TCID_50_, confluent MDCK cells in 96-well plates were inoculated with 10-fold serial dilutions of sample (six replicates per dilution) in serum-free DMEM containing 1 μg/mL TPCK-trypsin. Plates were incubated at 37 °C in 5% CO_2_ for 72 h, and CPE was recorded daily. TCID_50_ titers were calculated using the Reed–Muench method and expressed as log_10_ TCID_50_/mL for nasal washes or log_10_ TCID_50_/g tissue for lung homogenates. For qPCR, total RNA was extracted from lung homogenates and reverse-transcribed. Real-time PCR was performed using a TaqMan assay with primers and probe targeting the influenza A M gene or B NP gene (Supplementary Table S2). A standard curve was generated from 10-fold serial dilutions of a virus stock of known titer (log_10_ PFU/mL), and test sample viral loads were interpolated from the standard curve and reported as log_10_ PFU/g.

#### 2.5.4. Lung Pathology Detection

Left lung tissue was fixed with 10% formalin, sectioned, and stained with HE. The scope of pneumonia, alveolar inflammation, and inflammation of bronchi and pulmonary arterioles were scored with reference to the standards in Table 3.

### 2.6. Data Analysis

Office Excel 2017 and GraphPad Prism 9.0 were used for data collation and statistical analysis. Data were expressed as mean ± SEM or GMT ± SD. One-way analysis of variance (One-way ANOVA), two-way analysis of variance (Two-way ANOVA), or t-test was used for intergroup comparisons; two-tailed t-test was used for comparisons between two groups. A *p*-value < 0.05 was considered statistically significant.

## 3. Results

### 3.1. Evaluation of Immunogenicity

Ferrets were assigned to five groups (n = 6 per group): normal (PBS only, unchallenged), model (PBS, challenged), commercial vaccine, low-dose CpG-TIV, and high-dose CpG-TIV.

#### 3.1.1. Specific Ig Antibody Titer

**Figure 1:**
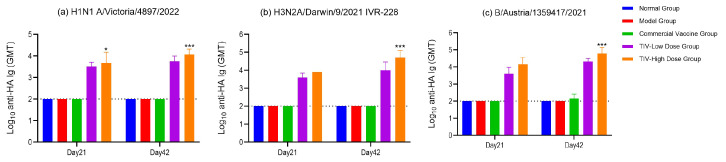
Log_10_-transformed geometric mean titers (GMT) of serum anti-HA Ig against three influenza strains at Day 21 and Day 42 post-vaccination. (**a**) A/Victoria/4897/2022 (H1N1); (**b**) A/Darwin/9/2021 IVR-228 (H3N2); (**c**) B/Austria/1359417/2021 (B/Victoria lineage). Dashed lines indicate the limit of detection (LOD). Two-way ANOVA analysis, * *p* < 0.05, *** *p* < 0.001 vs. Model Group.

On Day 21 and Day 42 post-immunization, peripheral blood was collected, and serum was isolated separately. Specific Ig antibodies against H1N1, H3N2, and BV were detected by indirect ELISA. No specific Ig antibodies against H1N1, H3N2, or BV were detected in the normal group or model group throughout the experiment; GMTs in both groups remained below the lower limit of detection (dashed line in Figure 1; LOD = 100), indicating that no specific antibodies were induced in the absence of immunization. In the commercial vaccine group, only an extremely low titer of BV-specific Ig antibody was detected on Day 42 (GMT = 144), while H1N1- and H3N2-specific antibodies remained undetectable (GMT < 100), suggesting that the commercial vaccine had extremely poor immunogenic efficacy and failed to effectively induce specific immune responses against the three tested influenza viruses in the body. In the low-dose and high-dose groups of the investigational trivalent vaccine, the GMT values of specific Ig antibodies against the three viruses all exhibited a time-dependent pattern, with those on Day 42 being significantly higher than those on Day 21. At the same time points (Day 21 and Day 42), the GMT values of specific Ig antibodies against the three viruses in the high-dose trivalent vaccine group were significantly higher than those in the low-dose group (*p* < 0.05), showing a clear dose-dependent effect. Additionally, there were differences in the immune induction efficacy of the vaccine on the three viruses, with an overall trend of BV > H3N2 > H1N1 (GMT values on Day 42 in the high-dose group: BV 60703 > H3N2 50564 > H1N1 11682). This indicated that the vaccine had the strongest immunogenicity against influenza B virus (BV), followed by H3N2, and relatively weak immunogenicity against H1N1, but it could induce high-titer specific antibodies against all three viruses (Figure 1).

These results demonstrate that the investigational CpG-TIV elicits robust, time- and dose-dependent specific Ig responses against H1N1, H3N2, and BV strains, with significantly stronger immunogenicity than the commercial vaccine. In comparison, the normal, model, and commercial vaccine groups do not induce effective specific humoral immunity.

#### 3.1.2. Hemagglutination Inhibition Titer

HAI assay results demonstrated that the normal and model groups exhibited undetectable HAI titers against all three vaccine-matched strains (H1N1, homologous H3N2, and B/Victoria) at both Day 21 and Day 42 post-immunization, with GMTs consistently below the lower limit of detection (LOD = 1:10), confirming that no strain-specific hemagglutination-inhibiting antibodies were generated without vaccination.

Compared with the model group, animals immunized with the commercial vaccine mounted only modest HAI responses against the three strains. GMTs in this group generally remained below 100 at Day 21 and declined further by Day 42, indicating limited immunogenicity and poor antibody persistence.

**Figure 2:**
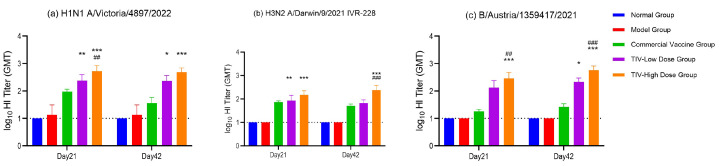
Serum hemagglutination inhibition (HAI) titers against three influenza strains. (**a**) A/Victoria/4897/2022 (H1N1); (**b**) A/Darwin/9/2021 IVR-228 (H3N2); (**c**) B/Austria/1359417/2021 (B/Victoria lineage). Dashed lines indicate the limit of detection (LOD). Data are presented as geometric mean titers (GMTs) with 95% confidence intervals. Statistical analyses were performed using two-way ANOVA. * *p* < 0.05, ** *p* < 0.01, *** *p* < 0.001 vs. Model Group; ## *p* < 0.01, ### *p* < 0.001 vs. CpG-TIV-Low-dose Group.

In contrast, both low- and high-dose formulations of the investigational CpG-adjuvanted trivalent influenza vaccine (CpG-TIV) induced markedly elevated HAI titers against all three vaccine-matched strains relative to the model and commercial vaccine groups (*p* < 0.05). GMTs in the low-dose CpG-TIV group exceeded those of the commercial vaccine by approximately 2- to 5-fold, and the high-dose group further increased titers by an additional 2- to 4-fold over the low-dose group across all strains and time points. A prominent dose-dependent effect was observed: HAI GMTs for all three strains were significantly higher in the high-dose CpG-TIV group than in the low-dose group at both Day 21 and Day 42 (*p* < 0.05, Figure 2). Detailed statistical comparisons and full numerical data are provided in Figure 2.

To evaluate baseline cross-reactive immunity against the antigenically drifted heterologous H3N2 strain A/Hong Kong/4801/2014, cross-HAI testing was performed on sera collected on Day 42 prior to viral challenge. Background correction was conducted using a baseline cutoff titer of 1:20 derived from negative control mice. Both raw uncorrected and background-corrected HAI geometric mean titers (GMTs) for all groups are listed in Table 4. After correction, the corrected HAI titers of all individual pre-challenge samples fell below the 1:20 cutoff. Consistently, the corrected GMTs of all groups were zero with a 0% seropositivity rate, verifying that none of the animals possessed pre-existing cross-reactive immunity against the challenge strain.

### 3.2. Validation of Challenge Model

**Figure 3:**
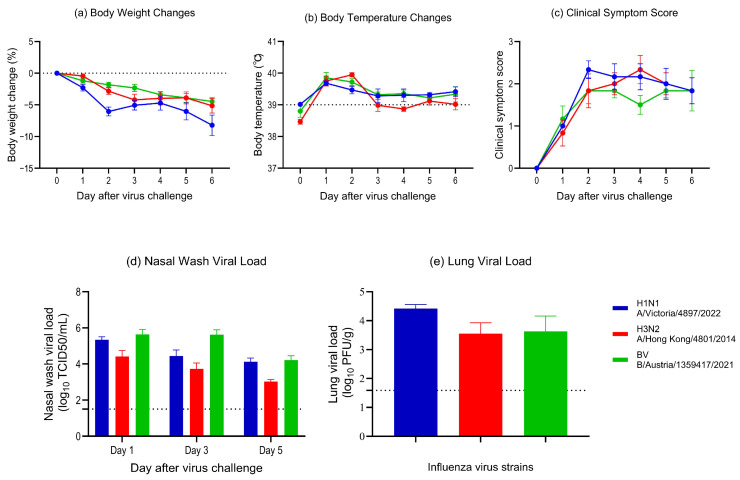
Clinical manifestations and viral loads of ferrets post-challenge with three influenza virus subtypes. (**a**) Percentage body weight change (%) relative to Day 0; (**b**) Rectal temperature; (**c**) Clinical symptom scores (nasal discharge, sneezing, vitality); (**d**) Nasal wash viral titers (log10 TCID50/mL) on Days 1, 3, and 5 post-challenge; (**e**) Pulmonary viral RNA loads quantified by absolute quantitative qPCR on Day 6 post-challenge. Horizontal dashed lines are displayed in panels (**a**,**b**,**d**,**e**): the horizontal dashed line in panel (**a**) denotes the zero-change baseline; the horizontal dashed line in panel (**b**) denotes the standard Recta temperature of 39 °C; the horizontal dashed lines in panels (**d**,**e**) denote the corresponding values of the Normal Group.

After challenge with the three subtypes of influenza viruses, ferrets in the model groups exhibited progressive body weight loss and elevated body temperature, with clinical symptoms mainly including nasal discharge, sneezing, and decreased vitality (Figure 3a–c). High viral titers were detected in nasal washes on Days 1, 3, and 5 post-challenge for all subtypes (Figure 3d). Regarding pulmonary viral load, conventional TCID_50_ assays detected no infectious live virus in the majority of lung tissues on Day 6, with only three animals in the BV group yielding positive results. However, using the more sensitive absolute quantitative qPCR method, significant viral RNA residues were still detectable in the lung tissues (Figure 3e). Collectively, these results demonstrate that ferrets are susceptible to all three influenza virus subtypes with evident upper respiratory tract viral shedding, confirming the successful establishment of the challenge model.

### 3.3. Evaluation of Challenge Protection Efficacy

#### 3.3.1. Body Weight, Body Temperature, and Clinical Symptom Scores

After challenge with H1N1, H3N2, or BV strains, ferrets in the model group showed significant weight loss, elevated body temperature, and typical influenza-like symptoms including nasal discharge, sneezing, and lethargy. CpG-TIV tended to improve these indicators in a dose-dependent manner, while the commercial vaccine showed no significant protective effects.

For the H1N1 A/Victoria/4897/2022 challenge, all CpG-TIV-vaccinated groups exhibited mild reductions in clinical scores, weight loss, and fever. The high-dose TIV group showed significant improvements in clinical symptoms, body weight, and temperature control compared with the model group.

For the heterologous H3N2 A/Hong Kong/4801/2014 challenge, the commercial vaccine did not improve clinical manifestations, weight, or temperature. In contrast, both low-dose and high-dose TIV groups showed reduced clinical scores, weight loss, and fever from Day 4 to Day 6 post-challenge, with the high-dose group presenting more pronounced improvements.

For the BV B/Austria/1359417/2021 challenge, neither the commercial vaccine nor the low-dose TIV group showed significant improvements. Only the high-dose TIV group exhibited markedly reduced clinical scores, alleviated weight loss, and improved temperature regulation.

**Figure 4:**
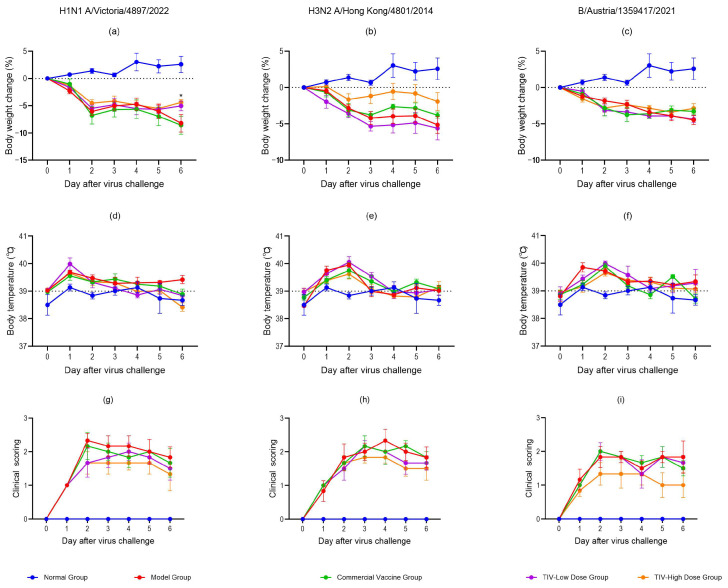
Dynamic changes in body weight percentage, rectal temperature, and clinical symptom scores of ferrets following viral challenge with three influenza strains. The first row (**a**–**c**) shows body-weight change, the second row (**d**–**f**) shows rectal temperature, and the third row (**g**–**i**) presents clinical-symptom scoring. Columns correspond to challenge models of H1N1 A/Victoria/4897/2022, H3N2 A/Hong Kong/4801/2014, and B/Austria/1359417/2021 (B/Victoria lineage). Group information is displayed in the figure legend at the bottom. Horizontal dashed lines in panels (**a**–**c**) indicate the zero-change baseline, while those in panels (**d**–**f**) indicate the standard rectal temperature of 39 °C.

Overall, the high-dose CpG-TIV most effectively alleviated influenza-related clinical symptoms, weight loss, and fever, whereas the commercial vaccine provided no significant protection in any challenge model. All results are shown in Figure 4.

#### 3.3.2. Viral Load in Nasal Lavage Fluid

After challenge, nasal lavage fluid was collected on Days 1, 3, and 5, and viral load was detected using the TCID_50_ method. The results are analyzed as follows. Compared with the normal group, the model group showed higher viral loads in the nasal wash fluid on Day 1, Day 3, and Day 5 after challenge, with statistical significance (*p* < 0.05). Meanwhile, the viral load presented a gradual decreasing trend, indicating that the challenge model was successfully established and the body could slowly clear part of the virus on its own, providing a reliable model for the subsequent evaluation of vaccine efficacy.

In the H1N1 A/Victoria/4897/2022 strain challenge model, compared with the normal group, the model group showed extremely high viral loads in nasal lavage fluid on Days 1, 3, and 5 (*p* < 0.001), with a gradual downward trend over time. This indicates that the viral infection model was successfully established and the virus in the respiratory tract could be naturally and gradually cleared. Compared with the model group, the commercial vaccine group showed no significant reduction in viral load in nasal lavage fluid at any time point, suggesting that it has no obvious promoting effect on the clearance of respiratory viruses induced by this strain. In contrast, the test trivalent vaccine group could significantly reduce the viral load on Days 3 and 5 (*p* < 0.01), and there was an obvious dose–response relationship between the vaccine dose and the antiviral effect.

For the challenge model of H3N2 A/Hong Kong/4801/2014 strain, compared with the model group, there was no significant difference in the viral load in the nasal wash fluid of the commercial vaccine group at different time points; the viral load in the nasal wash fluid of the low-dose group of the test trivalent vaccine showed a decreasing trend on Day 1, Day 3, and Day 5, among which there was a statistically significant difference in the reduction of viral load on Day 1 (*p* < 0.05); the viral load in the nasal wash fluid of the high-dose group of the test trivalent vaccine was significantly reduced on Day 1, Day 3, and Day 5 (*p* < 0.05).

For the challenge model of the Influenza B/Austria/1359417/2021 strain, compared with the model group, the commercial vaccine group showed no significant difference in the viral load in nasal lavage fluid on Days 1 and 3 after challenge, and a significant reduction was only observed on Day 5 (*p* < 0.05). This indicates that the commercial vaccine has limited antiviral effect against this strain, which can only slightly reduce the viral load in the late stage of infection and cannot achieve effective virus control throughout the whole process. For the high-dose and low-dose groups of the test trivalent vaccine, the viral load in nasal lavage fluid was significantly reduced on Days 1, 3, and 5 after challenge (*p* < 0.05); among them, the reduction amplitude of viral load was more obvious on Days 3 and 5 (*p* < 0.001), and on Day 5, the viral load in both the high-dose and low-dose groups of the trivalent vaccine was below the detection limit. This indicates that the test vaccine (regardless of high or low dose) has a clear antiviral effect against this influenza B strain, and its virus control effect is superior to that of the commercial vaccine.

**Figure 5:**
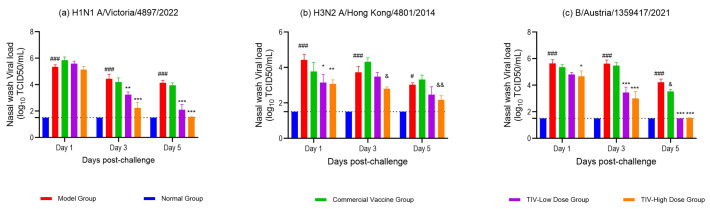
Log_10_-transformed viral loads (log_10_ TCID_50_/mL) detected in ferret nasal washes on Days 1, 3 and 5 post-challenge with three influenza strains. (**a**) H1N1 A/Victoria/4897/2022; (**b**) H3N2 A/Hong Kong/4801/2014; (**c**) B/Austria/1359417/2021 (B/Victoria lineage). Group information is shown in the legend on the right of the figure. Horizontal dashed lines denote the reference viral load value of the Normal Group in nasal wash samples. Statistical analyses were performed via two-way ANOVA; independent t-test was used for pairwise intergroup comparisons. Significance indicators: ### *p* < 0.001, # *p* < 0.05 vs. Normal Group; *** *p* < 0.001, ** *p* < 0.01, * *p* < 0.05 vs. Model Group; && *p* < 0.01, & *p* < 0.05 vs. Model Group.

Overall, the test trivalent vaccine can effectively inhibit the replication of viruses in the upper respiratory tract and accelerate viral clearance. Its antiviral effect is significantly superior to that of the commercial vaccine, and it shows a clear dose-dependent effect—the antiviral effect of the high-dose group is significantly better than that of the low-dose group, with a clear dose–response relationship (shown in Figure 5).

#### 3.3.3. Lung Virus Load

TCID_50_ results showed that no obvious viral load was detected in lung tissues of ferrets from G1–G4 groups following H1N1 A/Victoria/4897/2022 challenge. Similarly, undetectable pulmonary viral titers were observed in G5–G8 animals challenged with H3N2 A/Hong Kong/4801/2014. This phenomenon was attributed to robust spontaneous immune clearance of H1N1 and H3N2 viruses in ferrets during the late stage of infection, resulting in viral titers below the detection limit of the TCID_50_ assay.

**Figure 6:**
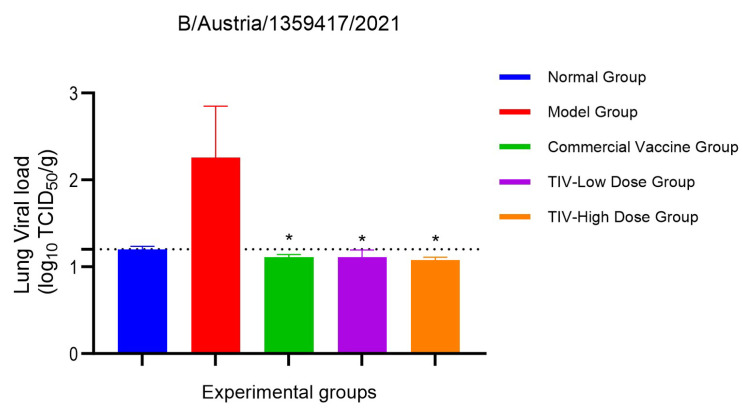
Results of viral load detection in lung tissues by TCID_50_ assay. Horizontal dashed lines denote the reference lung viral load value of the Normal Group. One-way ANOVA: * *p* < 0.05 vs. Model Group.

For the Influenza B/Austria/1359417 challenge, notable pulmonary viral loads were detected in 3 out of 6 ferrets in the G9 model group, whereas no detectable virus was found in the other individuals. In contrast, all three vaccine immunization groups exhibited markedly lower lung viral titers than the model group, with statistically significant differences (*p* < 0.05), demonstrating that vaccines effectively inhibited pulmonary viral colonization and persistent replication of influenza B virus (shown in Figure 6).

On Day 6 post-challenge, robust adaptive immune responses were fully established in ferrets, yet host endogenous immunity remained insufficient to completely clear the H1N1 A/Victoria/4897/2022 virus. Given the relatively low sensitivity of the conventional TCID_50_ assay and its inability to distinguish intergroup discrepancies at the late infection phase, absolute quantitative real-time PCR was adopted for accurate quantification of pulmonary viral nucleic acid copies. CT values of naive control ferrets remained stable at baseline background levels, and no specific viral nucleic acids were detected, which excluded nonspecific background interference.

In H1N1-challenged ferrets, the model group (G1) exhibited high levels of residual pulmonary viral loads, confirming successful and stable establishment of influenza pulmonary infection models. No statistical difference in viral load was observed between the commercial vaccine group (G2) and the PBS model group, indicating negligible pulmonary protective immunity and poor inhibitory effects on viral pulmonary replication. In contrast, both low- and high-dose investigational trivalent vaccines exerted prominent antiviral efficacy. Most samples from the low-dose group presented CT values > 35, while the majority of high-dose specimens were below the assay detection limit. Pulmonary viral loads were significantly reduced by more than 2 log_10_ in both vaccinated groups (*p* < 0.001), with clear dose-dependent protective effects and markedly superior pulmonary protection in the high-dose regimen.

For the H3N2 A/Hong Kong/4801/2014 challenge, host immune responses effectively cleared pulmonary viruses during the late infection stage. No hemagglutination-positive wells were observed across all serial dilutions, and TCID_50_ titers remained uniformly at background levels without distinguishable intergroup variations, suggesting that traditional virus isolation methods were not sensitive enough to reflect subtle differences in residual pulmonary virus. No significant differences were found between the commercial vaccine group and the model group, demonstrating that the commercial vaccine failed to suppress pulmonary H3N2 colonization, replication, or alleviate pulmonary viral burden. Combined with high-sensitivity qPCR results, both low- and high-dose trivalent vaccines significantly reduced intrapulmonary viral residues to near the detection threshold (*p* < 0.05), and exerted reliable dose-dependent pulmonary protective efficacy against H3N2 infection.

Regarding the Influenza B/Austria/1359417 challenge, persistently high viral titers were detected in lung tissues of the model group (G9). Hemagglutination-positive results were observed in 3 out of 6 ferrets, with peak Log_10_ viral load up to 4.07. Obvious individual variability verified a stable influenza B pulmonary infection model, and host immunity could not achieve complete clearance of pulmonary influenza B virus.

**Figure 7:**
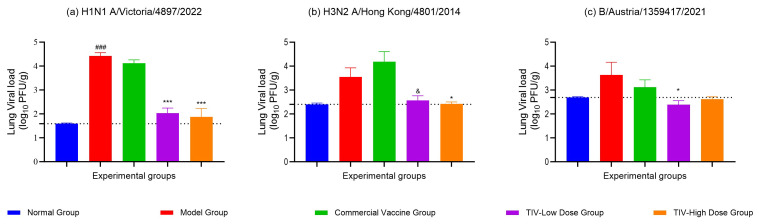
Results of viral load detection in lung tissues by qPCR assay. (**a**) Results of viral load detection in lung tissues by TCID_50_ assay in ferrets challenged with H1N1 A/Victoria/4897/2022; (**b**) Results of viral load detection in lung tissues by TCID_50_ assay in ferrets challenged with H3N2 A/Hong Kong/4801/2014; (**c**) Results of viral load detection in lung tissues by TCID_50_ assay in ferrets challenged with B/Austria/1359417/2021. Horizontal dashed lines denote the reference viral load values of the Normal Group. One-way ANOVA: ### *p* < 0.001 vs. Normal Group; *** *p* < 0.001 vs. Model Group, * *p* < 0.05 vs. Model Group; *T*-test: & *p* < 0.05 vs. Model Group.

Although no detectable hemagglutination-positive viruses were found in the commercial vaccine group (G10) by TCID_50_ detection, the vaccine failed to stably restrict long-term viral colonization and intrapulmonary persistent replication. No positive viral signals were detected in the low-dose trivalent vaccine group, with only minor random fluctuations in viral indicators. All samples from the high-dose vaccine group were below the TCID_50_ detection limit, showing powerful antiviral protection, complete elimination of pulmonary residual virus, and notably better pulmonary protective efficacy than commercial influenza vaccines (Figure 7).

#### 3.3.4. Lung Pathology Analysis

After separate challenge with three influenza virus strains (H1N1, H3N2, and BV), ferrets in the model groups developed varying degrees of pulmonary pathological damage compared with the normal group. Specifically, infection with H1N1 or influenza B virus resulted in significantly expanded inflammatory lesions and increased inflammatory cell infiltration scores around alveoli, bronchi, and pulmonary arterioles (*p* < 0.05 or *p* < 0.01). In the H3N2 challenge model, the lesion scope in ferrets was also significantly increased (*p* < 0.05), accompanied by elevated inflammatory infiltration around alveoli and blood vessels. These results confirmed that all three strains reliably induced typical pulmonary inflammatory lesions in ferrets, validating a suitable model for further vaccine efficacy evaluation.

In all challenge models, the commercial vaccine showed no significant improvement in any pulmonary pathological index in ferrets. It exerted limited effects on alleviating lung inflammation caused by the tested strains and failed to provide sufficient local respiratory tract protection. In contrast, the investigational trivalent vaccine showed significant dose-dependent pulmonary protection, with the high-dose formulation being markedly superior to the low-dose one.

**Figure 8:**
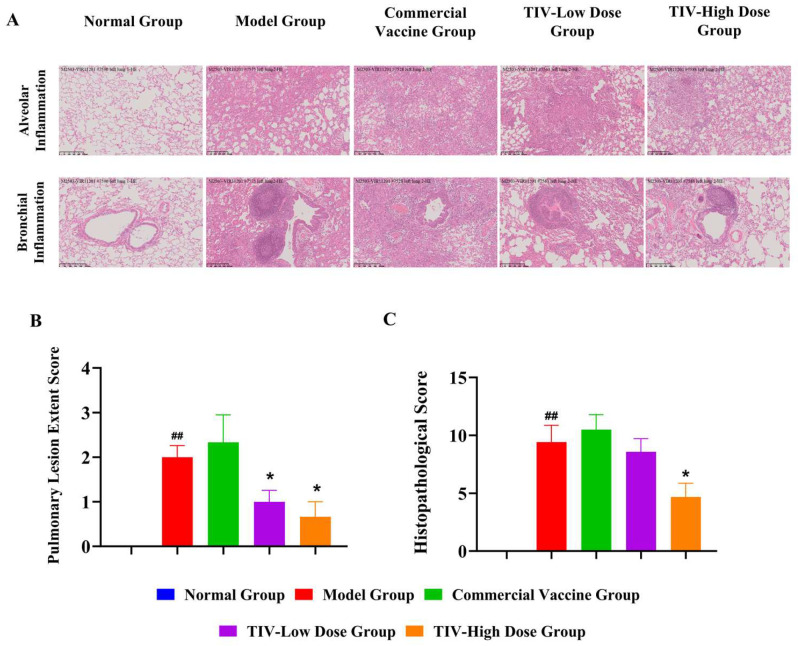
Dose-dependent protective effects of an investigational CpG-TIV against H1N1-induced pulmonary inflammation. (**A**) Lung hematoxylin-eosin (HE) staining analysis (H1N1 Challenge); (**B**) Pulmonary lesion extent score; (**C**) Histopathological score. *T*-test: ## *p* < 0.01 vs. Normal Group; * *p* < 0.05 vs. Model Group.

**Figure 9:**
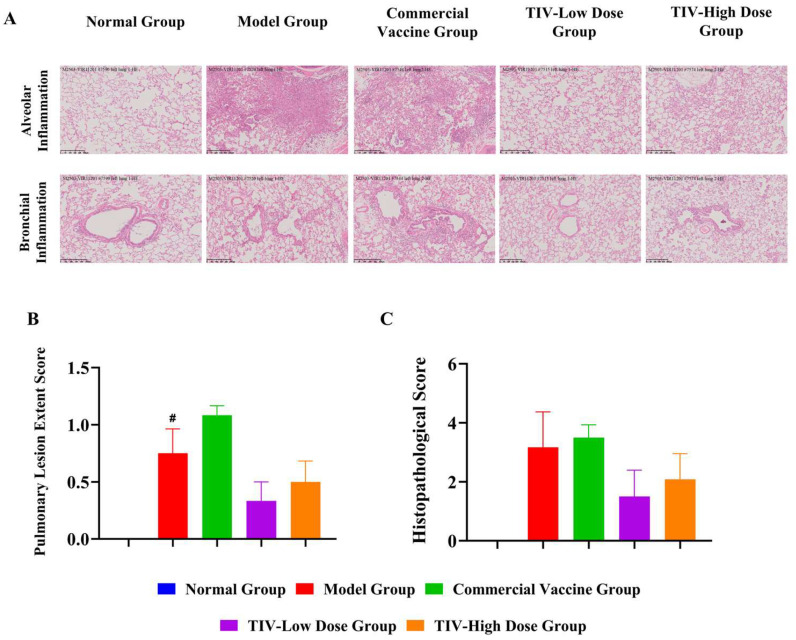
Dose-dependent protective effects of an investigational CpG-TIV against H3N2-induced pulmonary inflammation. (**A**) Lung hematoxylin-eosin (HE) staining analysis (H3N2 Challenge); (**B**) Pulmonary lesion extent score; (**C**) Histopathological score. *T*-test: # *p* < 0.05 vs. Model Group.

**Figure 10:**
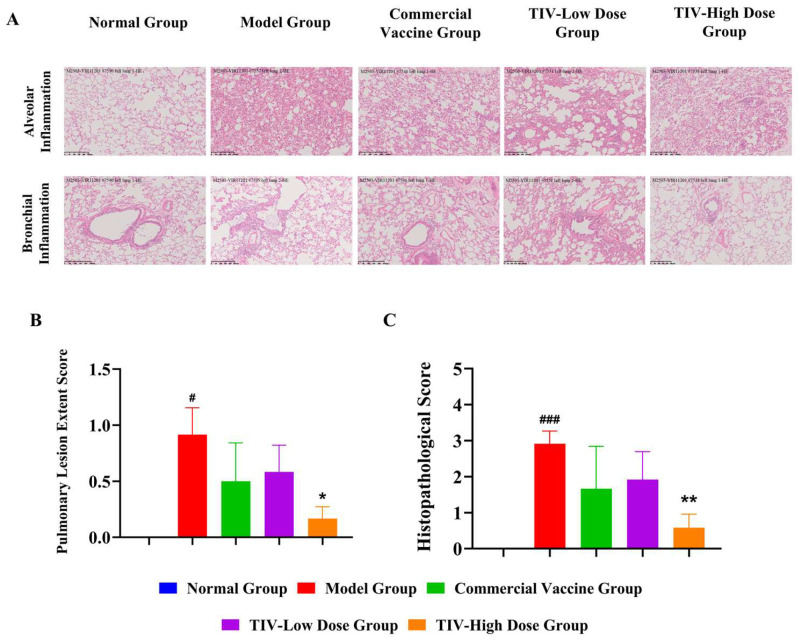
Dose-dependent protective effects of an investigational CpG-TIV against BV-induced pulmonary inflammation. (**A**) Lung hematoxylin-eosin (HE) staining analysis (BV Challenge); (**B**) Pulmonary lesion extent score; (**C**) Histopathological score. *T*-test: ### *p* < 0.001 vs. Normal Group; # *p* < 0.05 vs. Normal Group; ** *p* < 0.01 vs. Model Group; * *p* < 0.05 vs. Model Group.

H1N1 challenge model: The low-dose group of the trivalent vaccine could only significantly reduce the range of inflammatory lesions in lung tissue (*p* < 0.05), but had no significant improvement effect on inflammatory cell infiltration around alveoli, bronchi, and blood vessels. The high-dose group could simultaneously significantly reduce the degree of pulmonary lesions and the level of inflammatory cell infiltration in multiple sites, showing a comprehensive and effective overall anti-inflammatory protective effect (shown in Figure 8). H3N2 challenge model: After intervention with low and high doses of the trivalent vaccine, the indicators of pulmonary pathological damage and inflammatory infiltration in ferrets all showed a benign improvement trend, indicating that the vaccine had a potential protective effect against lung tissue damage induced by H3N2 infection (shown in Figure 9). BV challenge model: The low-dose group of the trivalent vaccine could only slightly alleviate pulmonary pathological damage without a statistically significant difference (shown in Figure 10). The high-dose group markedly alleviated inflammatory lesion extent and infiltration scores in lung tissues, with persistent anti-inflammatory and lung-protective effects.

### 3.4. Cross-Protective Effect

The test CpG-adjuvanted TIV does not contain the HA antigen of the heterologous drifted strain A/Hong Kong/4801/2014 (H3N2). Nevertheless, it significantly reduced weight loss, nasal and pulmonary viral loads, and lung pathological damage in ferrets compared with the model group (*p* < 0.05). In contrast, the commercial vaccine showed no significant protective effect against this heterologous H3N2 challenge. These results indicate that the CpG-adjuvanted TIV elicits effective cross-protection against antigenically drifted H3N2 virus, which may be associated with the induction of cross-reactive antibodies and cellular immune responses.

## 4. Discussion

This study systematically evaluated the immunogenicity and protective efficacy of the CpG-adjuvanted trivalent inactivated influenza vaccine (CpG-TIV) in a ferret model. The results showed that this candidate vaccine induced high levels of specific IgG antibodies and hemagglutination inhibition antibodies after immunization. Antibody levels increased significantly in a dose-dependent manner, indicating excellent immunogenicity. In challenge protection assays, the vaccine effectively alleviated clinical symptoms in ferrets, reduced viral loads in nasal washes and lung tissues, and mitigated inflammatory damage in the lungs. The high-dose group exhibited significantly better protection than the low-dose group, confirming substantial protective efficacy against H1N1, H3N2, and BV influenza viruses. Notably, the vaccine also provided effective cross-protection against the H3N2 A/Hong Kong/4801/2014 strain, which does not share the corresponding HA antigen. This effect may be related to cross-reactive antibodies or cellular immune responses induced by the vaccine, providing new insights for the development of broad-spectrum influenza vaccines.

The CpG-adjuvanted trivalent influenza vaccine constructed in this study presents prominent core advantages. It not only achieves potent dose-dependent protection but also elicits effective cross-protection against heterologous drifted strains, with overall protective performance superior to commercially available traditional unadjuvanted influenza vaccines. Most previously reported adjuvanted influenza vaccines show limited cross-protective efficacy and fail to provide effective defense against heterologous H3N2 variants. In contrast, through the synergistic strategy of CpG adjuvant combined with dosage optimization, this study achieved a breakthrough in robust cross-protection against the heterologous H3N2 A/Hong Kong/4801/2014 strain. The cross-reactive IgG antibody titer was significantly higher than that of commercial conventional trivalent influenza vaccines (*p* < 0.05). These findings provide new evidence for adjuvant selection and dose optimization in the design of broad-spectrum influenza vaccines.

Of note, the cross-protective potential of this candidate vaccine distinguishes it from conventional trivalent influenza vaccines. A recent study published in *Vaccines* confirmed that conventional unadjuvanted TIVs only induce strain-specific protective immunity without significant cross-reactivity against heterologous H3N2 variants. Other studies have also reported that some adjuvanted TIVs confer limited cross-protection, with cross-reactive antibody titers against heterologous H3N2 strains failing to reach protective thresholds. In sharp contrast, the CpG-adjuvanted vaccine in this study achieved reliable cross-protection against heterologous H3N2 strains through the synergy of adjuvant effect and dose optimization. It demonstrates important clinical value in addressing antigenic drift of influenza viruses and can serve as a preferred strategy for the prevention and control of antigenically mismatched seasonal influenza.

This study has certain limitations. First, cross-protective efficacy was only verified against a single heterologous H3N2 strain (A/Hong Kong/4801/2014). Future studies should expand the panel of challenge viruses to include more circulating H3N2 variants, such as those from the 2023–2024 influenza season, to further confirm the broad-spectrum protective potential of the vaccine. Second, the underlying immunological mechanisms remain incompletely elucidated. This study focused on humoral immune indicators including IgG and HAI antibodies, but did not deeply characterize the critical roles of virus-specific CD8^+^ cytotoxic T lymphocytes (CTLs), CD4^+^ Th1/Th2 cells, and other T cell subsets in viral clearance and long-term immunity. Subsequent work will use flow cytometry to analyze T cell subset profiles and cytokine secretion patterns to further clarify the coordinated mechanisms of humoral and cellular immunity in vaccine-induced protection.

In summary, this study adopted a multi-index, multi-dimensional evaluation system to comprehensively verify the immune-protective efficacy of the CpG-adjuvanted trivalent influenza vaccine, providing reliable experimental data to support subsequent clinical research and translational application of this candidate vaccine.

Although serum was collected at 6 dpi and archived, antibody responses at this early post-challenge time point were not assessed in the current study. Given that robust viral replication and clinical illness were already confirmed, evaluation of anamnestic antibody responses at later convalescent time points will be a valuable addition in future investigations to further characterize the memory B cell response induced by CpG-TIV.

## 5. Conclusions

In summary, the present study systematically evaluated the immunogenicity and protective efficacy of a CpG-adjuvanted trivalent inactivated influenza vaccine (CpG-TIV) in ferrets. The candidate vaccine induced robust, dose-dependent homologous protective efficacy against H1N1 A/Victoria/4897/2022, H3N2 A/Darwin/9/2021 IVR-228, and B/Austria/1359417/2021 strains, as well as significant cross-protection against the heterologous drifted H3N2 variant A/Hong Kong/4801/2014. High-dose CpG-TIV exhibited superior protective effects, markedly reducing clinical symptoms, body weight loss, nasal and pulmonary viral loads, and lung pathological injury compared with the commercial unadjuvanted vaccine. Notably, high-dose CpG-TIV reduced H1N1 viral load by up to 2.58 log10 at 5 days post-challenge.

These findings demonstrate that the combination of CpG adjuvant and optimized high-dose antigen formulation effectively broadens protective coverage against antigenically drifted influenza viruses, overcoming the limited cross-protection of conventional vaccines. This study provides rigorous preclinical evidence supporting the further clinical development and rational dose optimization of CpG-adjuvanted TIV as a broad-spectrum influenza vaccine to address seasonal influenza outbreaks and potential antigenic mismatches.

## Figures and Tables

**Table 1:** Experimental Grouping.

Group	Immunogen	Challenge Strain	Number of Ferrets (n)
G1-Model Group	PBS	A/Victoria/4897/2022 (H1N1)	6
G2-Commercial Vaccine Group	Commercial Vaccine		6
G3-CpG-TIV-Low-dose Group	Low-dose test batch HP-3001		6
G4-CpG-TIV-High-dose Group	High-dose test batch HP-3001		6
G5-Model Group	PBS	A/Hong Kong/4801/2014 (H3N2)	6
G6-Commercial Vaccine Group	Commercial Vaccine		6
G7-CpG-TIV-Low-dose Group	Low-dose test batch HP-3001		6
G8-CpG-TIV-High-dose Group	High-dose test batch HP-3001		6
G9-Model Group	PBS	B/Austria/1359417/2021 (B/Victoria lineage)	6
G10-Commercial Vaccine Group	Commercial Vaccine		6
G11-CpG-TIV-Low-dose Group	Low-dose test batch HP-3001		6
G12-CpG-TIV-High-dose Group	High-dose test batch HP-3001		6
G13-Normal Group	PBS	No challenge	3

**Table 2:** Clinical Symptom Scoring Criteria.

Score	Activity Status	Respiratory Symptoms
0	Alert and active	No symptoms
1	Alert, active when stimulated	Mild nasal discharge, occasional sneezing, or nasal congestion
2	Neither alert nor active	Profuse nasal discharge, frequent sneezing, or difficulty breathing

**Table 3:** Evaluation Criteria for Lung Hematoxylin-Eosin (HE) Staining.

Score	Pneumonia Extent Score
0	No lesions
1	Inflammation extent < 10%
2	Inflammation extent 11–25%
3	Inflammation extent 26–50%
4	Inflammation extent 51–75%
5	Inflammation extent > 75%
Score	Alveolar Inflammation (Hemorrhage + Exudation + Inflammation Severity + Alveolar Wall Thickness)
0	None
1	Mild
2	Moderate
3	Severe
Score	Bronchial and Pulmonary Arteriole Inflammation
0	None
1	Mild
2	Moderate
3	Severe

**Table 4:** Raw and background-corrected cross-reactive HAI GMTs against A/Hong Kong/4801/2014 in pre-challenge serum samples collected on Day 42.

Group	Raw HAI GMT	Corrected HAI GMT
Normal Group	17	0
Model Group	13	0
Commercial Vaccine Group	10	0
TIV-Low Dose Group	17	0
TIV-High Dose Group	17	0

## Data Availability

The data presented in this study are available on request from the corresponding author.

## Supplementary Materials

The following supporting information can be downloaded at: https://www.mdpi.com/article/10.3390/vaccines14070615/s1, Table S1: Key reagents and consumables. Table S2: Key instruments and equipment.

## Abbreviations

The following abbreviations are used in this manuscript:

CpG	Cytosine-phosphate-guanine oligodeoxynucleotide
ELISA	Enzyme-linked immunosorbent assay
PBS	Phosphate-buffered saline
GMP	Geometric Mean Titer
HE	Hematoxylin-Eosin staining
TCID50	50% tissue culture infective dose
