# Supplementary material for: Evaluation of Immunogenicity and Cross-Protective Efficacy of a CpG-Adjuvanted Trivalent Inactivated Influenza Vaccine in Ferrets
Source: Vaccines (Basel). 2026 Jul 14;14(7):615. doi: 10.3390/vaccines14070615 (PMC13418998; doi:10.3390/vaccines14070615)
Supplement: Supplementary file 1 [file vaccines-14-00615-s001.zip › vaccines-4383717-supplementary.pdf]

Supplementary Table S1. Key reagents and consumables.

Name	Catalog Number	Manufacturer
DMEM Medium	C11995500BT	Gibco
0.5M HEPES	KL10287RY	Kanglang Biology
RDE (Receptor Destroying Enzyme)	340122	National Institute of Infectious Diseases (Japan)
Fetal Bovine Serum	BC-SE-FBS01	Bio-Channel
0.25% Trypsin-EDTA	25200-072	Gibco
20% Turkey Red Blood Cells	KW-0120R-20	Kewei Biology
1% Guinea Pig Red Blood Cells	SBJ-RBC-GP001	Senbeijia
Penicillin-Streptomycin Solution	E607011-0100	Sangon Biotech (Shanghai) Co., Ltd.
Ficoll-Paque™ PREMIUM	17544203	Cytiva
Serum-Free Cell Cryopreservation Medium	C40100	Xinsaimai Biotechnology Co., Ltd.
1×PBS Buffer	BL302A	Biosharp
RNA Easy Fast Total RNA Extraction Kit for Animal Tissues/Cells	DP451	TIANGEN
TPCK Trypsin	T1426-50MG	Sigma
Bovine Serum Albumin	B2064	Sigma
PrimeScript™ RT reagent Kit (Perfect Real Time)	RR037A	Takara
Hieff Unicon qPCR TaqMan MIX	11205ES08	Yeasen
Goat Anti-Ferret Ig G H&L (HRP)	ab112770	Abcam
ELISA Coating Buffer (10×Coating Solution)	abs9290	Absin
ELISA Supplemental Solution Set	SEKCR02l	Sino Biologica

Supplementary Table S2. Key instruments and equipment.

**Instrument Name**	**Model**	**Manufacturer**
Biosafety Cabinet	BSC1300-Ⅱ-A2	Shandong Xinhua
Biosafety Cabinet	BSC1604-Ⅱ-A2	Sujing Antai
Inverted Microscope	CKX53	Olympus
Electric Pipette	YZ216AE0007879	Dragon Lab
Centrifuge	Centrifuge 5424	Eppendorf
Low-Speed Benchtop Centrifuge	TDZ5-WS	Hunan Xiangyi
Electric Constant Temperature Water Bath	HWS-12	Shanghai Yiheng Scientific Instruments

**Instrument Name**	**Model**	**Manufacturer**
Vertical Autoclave	LMQ.C	Shandong Xinhua
Homogenizer	PRO 200	PRO Scientific
-80℃ Ultra-Low Temperature Freezer	DW-86L348	Aucma
CO_2 Incubator	3111	Thermo
Multifunctional Microplate Reader	Varioskan LUX	Thermo
Electric Blast Drying Oven	DHG-9055A	Shanghai Yiheng
PCR Instrument	T100	Bio-Rad
Real-Time Fluorescent Quantitative PCR Instrument	CFX duet	Bio-Rad
Ultraviolet-Visible Spectrophotometer (Nanodrop)	Nanodrop One	Thermo
